# Genome-Wide Single Nucleotide Polymorphism Discovery and the Construction of a High-Density Genetic Map for Melon (*Cucumis melo* L.) Using Genotyping-by-Sequencing

**DOI:** 10.3389/fpls.2017.00125

**Published:** 2017-02-06

**Authors:** Che-Wei Chang, Yu-Hua Wang, Chih-Wei Tung

**Affiliations:** ^1^Department of Agronomy, National Taiwan UniversityTaipei, Taiwan; ^2^Crop Science Division, Taiwan Agricultural Research Institute, Council of AgricultureTaichung, Taiwan

**Keywords:** melon, genotyping-by-sequencing, imputation, single nucleotide polymorphism, genetic map, quantitative trait loci mapping

## Abstract

Although genotyping-by-sequencing (GBS) enables the efficient and low-cost generation of large numbers of markers, the utility of resultant genotypes are limited, because they are enormously error-prone and contain high proportions of missing data. In this study, we generated single nucleotide polymorphism (SNP) markers for 109 recombinant inbred lines of melon (*Cucumis melo* L.) using the GBS approach and ordered them according to their physical position on the draft double haploid line DHL92 genome. Next, by investigating associations between these SNPs, we discovered that some segments on the physical map conflict with linkage relationships. Therefore, to filter out error-prone loci, 4,110 SNPs in which we have a high degree of confidence were selected as anchors to test independence with respect to unselected markers, and the resultant dataset was then analyzed using the Full-Sib Family Haplotype (FSFHap) algorithm in the software TASSEL 5.2. On the basis of this analysis, 22,933 loci that have an average rate of missing data of 0.281% were used to construct a genetic map, which spans 1,088.3 cM across 12 chromosomes and has a maximum spacing of 6.0 cM. Use of this high-quality linkage map enabled the identification of several quantitative trait loci (QTL) known to control traits in fruit and validated our approach. This study highlights the utility of GBS markers for the identification of trait-associated QTLs in melon and facilitates further investigation of genome structure.

## Introduction

Melon (*Cucumis melo* L., 2*n* = 24) is an economically very important fruit crop. About 1.1 million hectares of melon are grown around the world, generating about 1.6 billion US dollars in export value, making this fruit the second most important member of the Cucurbitaceae family (FAOSTAT, 2013). In addition, the nutritional quality of melon is high, as these fruits are low in calories and rich in nutrients including potassium, vitamin C, and β-carotene (Lester, 1997). While it is debated whether melons first evolved in Africa (Kerje and Grum, 2000) or in Asia (Sebastian et al., 2010), this fruit is highly diverse in morphology and biochemistry (Stepansky et al., 1999) and is classified into two subspecies, *melo* and *agrestis* (Jeffrey, 1980). The first of these subspecies comprises ten botanical groups while the second is composed of five (Pitrat, 2008); most commercial melon cultivars belong to the *melo* subspecies, including *cantalupensis*, *inodorus*, and *reticulatus*. A large volume of research to date has been dedicated to dissecting the genetic architecture of the fruit traits in melon that are related to quality and consumer acceptance, including β-carotene concentration (Cuevas et al., 2008, 2009; Harel-Beja et al., 2010; Ramamurthy and Waters, 2015; Tzuri et al., 2015) and the morphology of fruit (Périn et al., 2002a; Fernandez-Silva et al., 2010; Harel-Beja et al., 2010; Díaz et al., 2014; Ramamurthy and Waters, 2015). A genetic linkage map is an important tool used to study the genetic architecture of traits and the genome structure of species. The first genetic map for melon was constructed in 1996 on the basis of a number of molecular markers, including restriction fragment length polymorphism, random amplified polymorphic DNA, and isozymes (Baudracco-Arnas and Pitrat, 1996). A number of linkage maps for melon have been constructed to date encompassing a few 100s markers (Oliver et al., 2001; Périn et al., 2002b; Cuevas et al., 2008, 2009; Fernandez-Silva et al., 2008; Fukino et al., 2008; Deleu et al., 2009; Fang et al., 2009; Boissot et al., 2010; Harel-Beja et al., 2010); eight of these maps were used as the basis for a consensus genetic map 1,150 cM in size that comprised 1,592 markers and was constructed to improve resolution and facilitate comparisons (Diaz et al., 2011). By increasing marker density, the precision of quantitative trait loci (QTL) identification and estimates of their effects can be improved and used to potentially distinguish between closely related loci (Stange et al., 2013). In addition, connecting physical and high-density genetic maps enables more precise positional detection of recombination breakpoints (Yu et al., 2011) and the identification of hot and cold recombination spots (Spindel et al., 2013).

The advent of next generation sequencing (NGS) technology has enabled the generation of enormous volumes of DNA sequences over short timescales at low cost. Indeed, the cost of NGS sequencing can be reduced even further by employing reduced representation and multiplex sequencing strategies, enabling multiple samples to be sequenced simultaneously. These advances mean that sequencing-based genotyping approaches are now attractive research tools.

We know that the size of the melon genome is about 450 Mb (Arumuganathan and Earle, 1991); it has been sequenced and assembled *de novo* into the 375 Mb draft DHL92 genome (Garcia-Mas et al., 2012). Recently, the quality of this draft genome was significantly improved on the basis of a high-resolution genetic map that employed 580 SNPs to anchor 354.8 Mb sequences enabling 325.4 Mb of this assembly to be oriented (Argyris et al., 2015). To facilitate subsequent integration of information from previous genetic studies into the melon draft genome, 836 genetic markers including simple sequence repeats (SSR) and SNPs from the consensus map generated by the International Cucurbit Genomic Initiative were mapped onto the melon draft genome version CM_3.5.1 (Diaz et al., 2015). These genetic and genomic resources have paved the way for the development of high-resolution marker maps in melon.

Genotyping-by-sequencing (GBS) is one of a low cost and high-throughput sequencing-based genotyping approach that utilizes a relatively simple library construction protocol and efficiently generates tens of thousands of genome-wide markers (Elshire et al., 2011). Markers resulting from GBS have been widely used in genetic map construction including mapping QTLs in plant species that lack a high-quality reference genome, for example oat (Huang et al., 2014), apple (Gardner et al., 2014), peach (Bielenberg et al., 2015), cabbage (Lee et al., 2016), pumpkin (Zhang et al., 2015), and blueberry (McCallum et al., 2016). However, the disadvantages of GBS include the generation of error-prone genotypes and a high rate of missing data due to low coverage sequencing, tradeoffs against the pooling of large sample numbers to reduce cost. Thus, in order to effectively use genome-wide markers produced from GBS platforms in downstream applications, quality control of marker genotypes is required. This can be achieved by implementing a series of bioinformatics pipelines including SNP calling, error correction, and the use of imputation algorithms to analyze raw data, leading to the production of high-quality and high-density genetic maps with low investment (Spindel et al., 2013; Edae et al., 2015).

We applied the GBS approach in this study to sequence 109 melon recombinant inbred lines and their two parents, in conjunction with use of data quality control and imputation workflows. We generated a set of high-density GBS markers that were used to construct a high-quality genetic map and to identify with high resolution trait-controlling QTLs in melon. We hope that our results will demonstrate that GBS can be used as a powerful tool to accelerate the process of molecular breeding in melon.

## Materials and Methods

### Melon Recombinant Inbred Lines Mapping Population

A mapping population of 109 RILs in F7 generation derived from a cross between the TARI-08874 (*Cucumis melo* ssp. *melo*) and “Bai-Li-Gua” (*C. melo* ssp. *agrestis*) cultivars was used for SNP genotyping. Total genomic DNA (gDNA) was extracted from healthy young leaves using a Qiagen Plant DNeasy kit and was stored at –20°C prior to preparation of a sequencing library.

### GBS and SNP calling

We prepared a sequencing library by following the GBS protocol developed by Elshire et al. (2011). Briefly, 100 μg of genomic DNA from each inbred line was digested using the ApeKI four base cutter and ligated with a unique barcode-adaptor for polymerase chain reaction amplification. Each 96-plexed barcode-labeled amplicon library was sequenced in one lane on an Illumina HiSeq 2500 machine.

Single-end reads (ca. 125 bp) in FASTQ format were then processed using the GBS pipeline in the TASSEL 3 software (Glaubitz et al., 2014) on Linux operating system. Melon genome DHL92 assembly v3.5.1 pseudomolecules (CM3.5.1_pseudomol.fa) were downloaded from MELONOMICS^1^ and used as references for the alignment of sequenced reads.

### Construction of Linkage Groups and Validation of Marker Orders Using SNPs

This set of 4,164 markers was analyzed by calculating pairwise logarithm of odds (LOD) scores and recombination fractions using the software R/qtl (Broman et al., 2003). Markers were clustered in one group when their LOD scores exceeded 10.

A subset of 4,110 selected markers were then used to construct a reference-based genetic map based on their physical positions and a *de novo* genetic map that was constructed using the minimum spanning tree (MST) method (Wu et al., 2008) in the ASMap package (Taylor and Butler, 2015). Genetic distances were estimated for both maps by using the Kosambi mapping function in the R/qtl software (Broman et al., 2003).

### Determination of Parental Genotypes

The set of 46,590 original SNPs was first filtered by applying a missing data rate of less than 90% and a minor allele frequency (MAF) greater than 5%. Next, to improve the call rate of the two parents, markers were imputed using the Fast, Inbred Line Library Imputation (FILLIN) method in the software TASSEL 5.2 (Swarts et al., 2014) with default setting applied. In this way, the missing genotypes of the two parents can be inferred based on the haplotypes of the entire RIL population. Parental genotypes were determined using the dataset imputed by the FILLIN algorithm while ambiguous markers (i.e., cases where the alleles from two parents cannot easily be discriminated) were removed from the dataset.

### RIL Genotype Filtering and Imputation

A series of data processing and filtering steps were applied using a customized R script (flow chart in Supplementary Figure S1). First, to detect and remove SNP markers that were apparently misallocated from the RILs dataset, we used the 4,110 markers from the previous step as anchors to detect error-prone loci. Fisher’s exact test was then applied to the pre-imputation dataset of 109 RILs to examine dependence between a marker and its flanking anchors. Thus, each marker was tested on the basis of its nearest four anchors; two before and two after each respective marker in the study. Each marker was regarded as error-prone and was discarded if any of its related anchor was found to be independent (*p* > 0.05).

Markers that passed Fisher’s exact test were then imputed using the FSFHap method in the TASSEL 5.2 software with default parameters applied (Swarts et al., 2014).

### Removal of Genotyping Errors Using a Sliding Window Approach

Because double recombination events within a small interval should occur rarely in a bi-parental population, the detection of double recombination events in a high-density marker genetic map is most likely the result of genotyping errors. To remove these potential errors, we applied a sliding window approach modified from the SMOOTH algorithm (van Os et al., 2005) in our custom R script (Supplementary Figure S2). Thus, a marker in the middle of a window was compared with its flanking markers to calculate a window score and to determine whether, or not, it represents a genotyping error. A window score was calculated by dividing the number of markers with a different genotype from the central marker by the number of markers in the window excluding the central one. Because just sites that are present are informative, all missing genotypes were ignored when this sliding window method was implemented (Supplementary Figures S2A,B).

Each window comprising 25 markers was subject to this analysis and scores greater than 0.8 were removed. However, note that the sliding window will skip in cases of markers on both ends of chromosomes as there are insufficient numbers of flanking markers (Supplementary Figure S2C); in other words, the first and last 12 markers on each chromosome could not be corrected using this approach. Subsequent to the removal of potential genotyping errors, loci with missing rates greater than 10% or MAF scores lower than 5% were discarded and the remainder used to construct a reference-based genetic map.

### Construction of a Reference-Based Genetic Map and Data Analysis

The segregation distortion of 22,933 imputed markers was determined by using a chi-square test with a 5% threshold following Bonferroni correction. Genetic map distances were measured using the Kosambi function in the R/qtl software (Broman et al., 2003), and recombination rate was calculated in each sliding window following genetic distance estimation. The recombination rate of a 1 Mb window was evaluated by regressing physical distances onto genetic distances for the markers within each window. For all the markers within a window, their coordinates on physical map were regressed onto the corresponding coordinates on genetic map, the slope of the simple linear regression was used to represent the recombination rate in the given window.

### QTL Mapping of Fruit Traits in Melon

Because of the genetic features of RILs, polymorphic markers were collapsed into recombination bins for QTL mapping. Thus, we defined each recombination bin as the collection of markers between two recombination breakpoints, although in some cases, markers in a region where a recombination breakpoint could not be determined were also considered to comprise a bin (Supplementary Figure S3). This process resulted in 2,493 bins spanning the population.

Quantitative trait loci mapping was then performed using the R/qtl software (Broman et al., 2003), capturing external color (EC), flesh color (FC), fruit diameter (FD), fruit length (FL), fruit maturity (FM), fruit thickness (FT), fruit weight (FW), net density (ND), net thickness (NT), pistil scar width (PSW), ratio of length and diameter (RLD), and soluble solid content (SSC). In addition, depending on trait, composite interval mapping (CIM) encompassing an appropriate number of covariates (i.e., between two and five markers) and a window size of 10 cM was implemented using a single-QTL model. The Haley–Knott regression (Haley and Knott, 1992) method with a 1 cM interval was used for CIM with the LOD threshold determined using 1,000 permutations (Churchill and Doerge, 1994) for false positive probability at α = 0.05. In this way, presence of a putative QTL was determined when the LOD score was higher than the threshold, while a 95% Bayes credible interval was applied to determine QTL interval. Additive effect and phenotypic variance explained by each QTL (R^2^) were estimated at the highest LOD peak; while 255 (10%) and 504 (20%) of the 2,493 total bins were randomly selected for CIM to simulate QTL mapping with relatively low-density markers. The same cofactor numbers previously used were implemented in the two subsets with reduced marker density; if QTLs detected within the 2,493 bins were not detected within these two subsets, then the number of cofactors was adjusted.

Genotype filtering, imputation, map construction and QTL analysis were performed on personal computer with Intel Core i5-2410M 2.3 GHz CPU and 8 Gb RAM.

## Results

### GBS and SNP Discovery

Two 96-plex ApeKI-digested libraries comprising 109 recombinant inbred lines and their parents, TARI-08874 (*Cucumis melo* ssp. *melo*) and “Bai-Li-Gua” (*C. melo* ssp. *agrestis*), were sequenced on two lanes of an Illumina HiSeq2500 machine. Each lane resulted in the generation of ca. 318 million short single-end reads, and a total of 4,687,134 unique tags (sequence reads) were obtained when the two lanes were combined using the TagCount and MergeMultipleTagCount functions in the GBS pipeline of the TASSEL 3 software (Glaubitz et al., 2014). Thus, by aligning unique tags to the DHL92 pseudomolecule sequence, the position of each was mapped and SNPs were identified. Following the removal of putative error-prone and spurious SNPs using population level genetic-filters, a total of 46,590 SNP sites were retained for downstream validation and processing.

### Improving the Accuracy and Quality of Anchor SNPs

**FIGURE 1:**
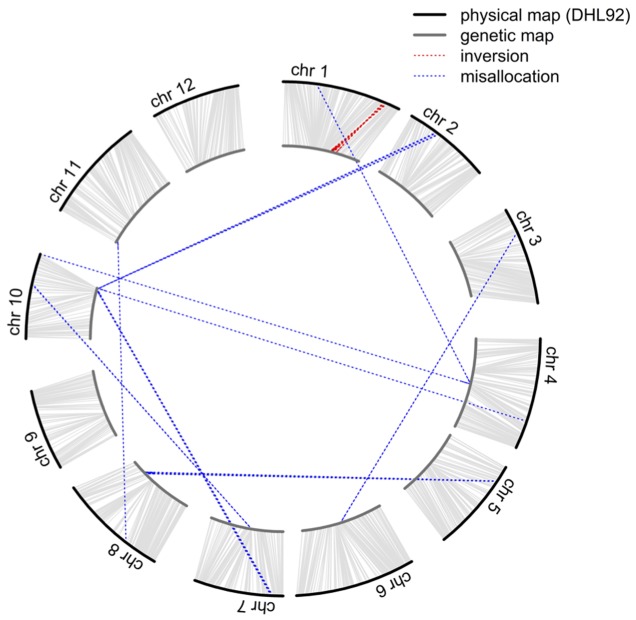
**Comparison of the order of 4,164 markers shared between the physical and genetic maps.** The outer circle represents the physical map while the inner circle represents the genetic map. Misallocations and inversions are highlighted by the blue and red dotted lines, respectively.

Although 46,590 SNP sites were identified in biparental RILs, the volume of missing data (ca. 45.9%) could significantly hinder application of this dataset, and the alignment of sequenced reads to the DHL92 draft genome (Garcia-Mas et al., 2012; Argyris et al., 2015) may also affect the accuracy of SNP position. Therefore, to improve marker quality and accuracy for QTL mapping, we initially investigated the linkage relationships of 4,164 selected SNPs that did not have any missing data in RILs as well as the two parents and calculated pairwise LOD scores and recombination fraction. While these 4,164 markers were classified into 12 linkage groups with LOD scores greater than 10, 54 could not be clustered correctly (**Figure 1** and Supplementary Table S1); these misallocated markers were removed and the remaining 4,110 used to construct a reference-based and a *de novo* map by ordering markers on the basis of the physical position of SNPs and the MST method (Wu et al., 2008), respectively. The reference-based and *de novo* maps encompassed 1,180.7 and 1,163.6 cM, respectively, and exhibited a high degree of shared marker order, with the exception of a region where 28 markers were inverted on chromosome 1 (**Figure 1** and Supplementary Figure S4), an approximately 1.1 Mb segment that spanned from 30,481,228 to 31,601,658 bp.

In order to confirm that misallocation of the 54 dropped and 28 inverted markers was not the result of BWA (Burrows-Wheeler Aligner) alignment errors during the SNP calling process, their tag sequences were searched against the DHL92 draft genome using the BLAST (Basic Local Alignment Search Tool) software (Camacho et al., 2009). Results show that 2,335 of the 2,684 unique tags in the 54 misallocated markers were successfully matched (*E*-value < 10^-5^) to the DHL92 draft genome, while comparisons of the physical positions of the 2,335 tags identified by the BLAST and BWA methods show that 2,278 (97.56%) could be aligned to either the same place or a surrounding region (±100 bp). In the case of the 28 inverted markers, 1,178 out of 1,383 unique tags were successfully matched to DHL92 draft genome sequences, with all tags aligned either to the same or a surrounding region (±100 bp). These results suggest that the alignment algorithm applied in the TASSEL-GBS SNP calling pipeline does not lead to the misallocation of markers.

### Genome-Wide SNP Filtering and Imputation

To further enhance the quality of our SNP markers, 46,590 were initially filtered on the basis of less than 90% missing data and a MAF greater than 5% in two parents and 109 RILs. Taking into account that the call rate of the two parents was just 70%, we imputed 27,505 markers from the parental and RIL genotypes using the FILLIN method in the TASSEL 5.2 software (Swarts et al., 2014). Following this process as well as the removal of monomorphic, ambiguous markers between the parental genotypes, we observed no further missing data in two parents and so the 25,578 markers were further imputed for RILs.

To improve the quality of our RIL genotypes, we employed 4,110 high-quality SNPs from our initial survey as anchors to detect error-prone markers in the set of 25,578 pre-imputation, and applied Fisher’s exact tests to examine the dependence between each and its flanking anchors. Thus, a total of 2,165 error-prone SNPs that failed this test were removed from the dataset, and the remaining 23,413 markers of 109 RILs were imputed using the FSFHap method (Swarts et al., 2014). Following imputation, the average missing data in RIL genotypes decreased from 44.49 to 1.04%.

We assessed the imputation accuracy of the FSFHap method by calculating the percentage of matched genotypic values between our imputed and original data at masked sites. Because as our plant materials were RILs, we were interested in imputation accuracy on homozygotes; thus, 5% of the total homozygous genotypes were randomly masked and then imputed using FSFHap. Ten replicates were performed on these masked datasets to calculate imputation accuracy; results show that average accuracy over ten replicates was 99.29% (**Table 1**).

**Table 1:** Imputation accuracy using the FSFHap method.

	Accuracy	Proportion of non-imputed genotype in masked sites
Rep1	0.9927	0.0402
Rep2	0.9931	0.042
Rep3	0.993	0.0467
Rep4	0.9926	0.1172
Rep5	0.9931	0.0536
Rep6	0.9931	0.0582
Rep7	0.9928	0.0649
Rep8	0.9931	0.0765
Rep9	0.9925	0.0842
Rep10	0.9927	0.091
Average ± *SD*	0.9929 ± 0.002	0.0675 ± 0.0247

*Accuracy was calculated as the proportion of the genotype imputed to the same genotype when the original was masked.*

### Construction of a Melon High-Density Linkage Map Using 22,933 Imputed Markers

**FIGURE 2:**
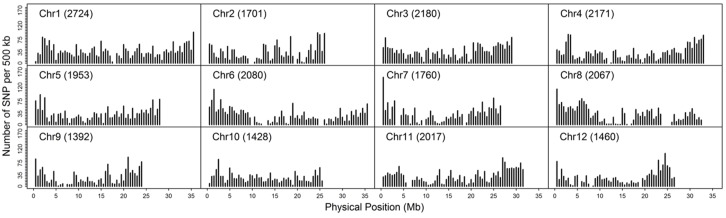
**Distribution of 22,933 imputed single nucleotide polymorphisms (SNPs) at 500 kb intervals on 12 chromosomes**.

**FIGURE 3:**
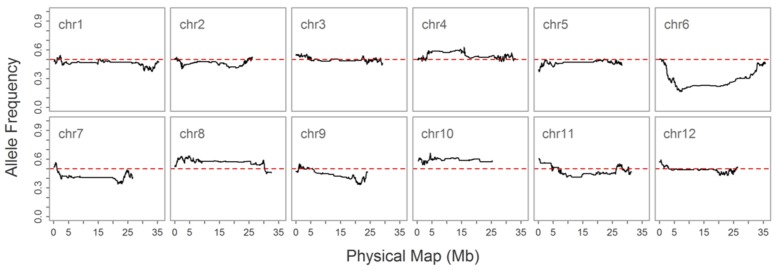
**Allele frequencies on 12 chromosomes in the “Bai-Li-Gua” cultivar.** The red dotted line denotes a frequency of 0.5

As double crossing-over within a small interval region is expected to be a rare event in a bi-parental population of RILs, occurrences in our dataset are probably the result of SNP calling errors and thus can potentially affect the size of a genetic map (Huang et al., 2009). Therefore, to detect and remove double crossing-over events, we applied a sliding window approach modified from the SMOOTH algorithm (van Os et al., 2005) to our dataset, removing 0.17% of data points and 480 markers that comprised more than 10% missing data. A total of 22,933 imputed and filtered markers were used to construct the final genetic map, missing data on the 12 chromosomes ranged between 0.169% (chromosome 5) and 0.43% (chromosome 11) at an average of 0.281%, and heterozygosity ranged between 0.336% (chromosome 9) and 1.947% (chromosome 10) with an average of 1.018%, as expected (**Table 2**). In order to investigate marker distribution, we counted the number of SNP markers in 500 kb intervals across the entire genome (**Figure 2**); results show that while some regions contain no markers, the highest density is seen at the top of chromosome 7 (i.e., 153 markers within a 500 kb region). We further examined the pattern of segregation distortion by performing a chi-square test on each marker; application of a 5% significance threshold level following Bonferroni correction, showed that 937 markers exhibit significant distortion and all were located on chromosome 6. These alleles were skewed toward the parent TARI-08874 with the exception of both ends of the chromosome where the maximum frequency of this allele was 0.83 (**Figure 3**).

**Table 2:** Summary data of 22,933 single nucleotide polymorphisms (SNPs) imputed across 12 chromosomes.

Chr	Heterozygous rate	Missing data rate	Proportion of non-imputed data	Proportion of imputed data
1	0.005509976	0.002536071	0.002512495	0.4655728
2	0.009325329	0.002777643	0.002664380	0.4184533
3	0.007932834	0.004225234	0.004208400	0.4573773
4	0.005278082	0.003570840	0.003511678	0.4162332
5	0.016516580	0.001686420	0.001639444	0.4452289
6	0.017157728	0.001795166	0.001764291	0.4001191
7	0.008913678	0.002074646	0.002017306	0.4422383
8	0.008539611	0.002205918	0.002152657	0.4491951
9	0.003367869	0.003242645	0.003209691	0.4142676
10	0.019466502	0.002576260	0.002467042	0.4601933
11	0.013954779	0.004302875	0.004239196	0.4321751
12	0.008225462	0.002576348	0.002532361	0.4225085
Total	0.010184034	0.002811141	0.002759534	0.4365637

**FIGURE 4:**
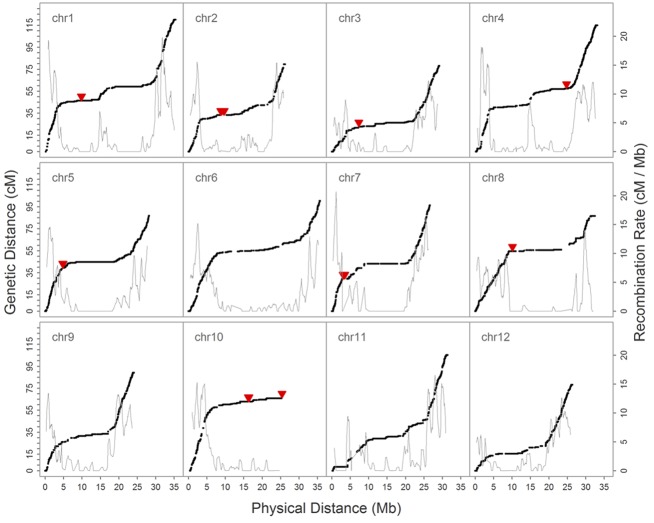
**Relationship between the genetic map and recombination rate (cM/Mb) on 12 chromosomes.** The black solid dot denotes the position of SNPs on genetic and physical maps, the solid red triangle marks the physical position of 54 misallocated SNPs, and the gray solid line shows the recombination rate.

We then constructed a genetic map encompassing 22,933 markers, including the inverted ones on chromosome 1, which spanned 1,101.6 cM and had a maximum gap of 8.8 cM on chromosome 1. A total of 2,247 recombination breakpoints were detected across 109 RILs, each of which had 20.61 breakpoints on average. We revealed hot spots and suppression of recombination by integrating information on genetic and physical distances (**Figure 4**); data show that the recombination rate was on average 3.13 cM/Mb across the 12 chromosomes, ranging between 2.66 cM/Mb (chromosome 10) and 3.70 cM/Mb (chromosome 9). The maximum calculated recombination rate was 20.76 cM/Mb, close to the end of chromosome 7 (**Figure 4**), and we also highlighted the physical positions of 54 misallocated markers, located near to, or at, regions of relatively low recombination (**Figure 4**).

Subsequent to correction of the marker order in the inverted segment on chromosome 1, genetic distance in the modified map decreased to 1,088.3 cM (i.e., chromosome 1 distance was reduced to 106.7 cM) while the maximum recorded gap was 6.0 cM on chromosome 4, not chromosome 1 (**Table 3**). Similarly, the number of recombination breakpoints was reduced to 2,223, an average of 20.39 per RIL line.

**Table 3:** Genetic map and recombination rate summary.

	Number of SNPs	Number of bins	Genetic distance (cM)	Maximum gap	Physical distance (bp)	Recombination rate (cM/Mb)
Chr 1	2,724	260 (263^∗^)	106.7 (120.0^∗^)	4.3 (8.8^∗^)	35,191,472	3.03 (3.41^∗^)
Chr 2	1,701	169	79.5	3.8	26,170,228	3.04
Chr 3	2,180	196	78.2	2.9	29,150,566	2.68
Chr 4	2,171	253	114.9	6.0	32,910,366	3.49
Chr 5	1,953	198	86.8	1.8	28,148,921	3.08
Chr 6	2,080	253	100.4	3.5	35,697,279	2.81
Chr 7	1,760	202	96.3	2.9	26,603,112	3.62
Chr 8	2,067	204	86.6	4.6	32,490,372	2.66
Chr 9	1,392	199	89.2	2.9	24,104,665	3.70
Chr 10	1,428	142	66.2	5.7	24,868,054	2.66
Chr 11	2,017	221	105.3	5.8	31,233,764	3.37
Chr 12	1,460	196	78.3	2.7	26,355,078	2.97
Total	22,933	2,493 (2,496^∗^)	1,088.3 (1101.6^∗^)			

*^∗^This number was calculated before the marker order of inversion segment on chromosome 1 was corrected.*

### Bin Selection and QTL Mapping of Fruit Traits in Melon

**FIGURE 5:**
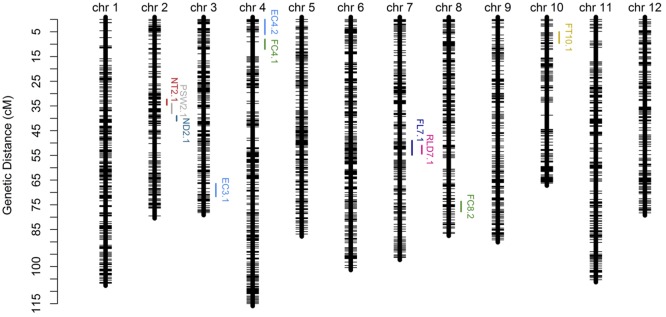
**Genetic map and quantitative trait loci (QTL) mapping results on the basis of 2,493 bins (i.e., the collection of 22,933 GBS markers).** Abbreviations: EC = external color; FL = fruit length; FT = flesh thickness; FC = flesh color; ND = net density; NT = net thickness; PSW = pistil scar width; RLD = ratio of length and diameter.

In order to verify the utility of the high-density map constructed using GBS markers, 2,493 bins were used to identify QTLs for twelve fruit traits. The results of this analysis show that ten QTLs were detected for eight traits (i.e., EC, FC, FL, FT, ND, NT, RLD, and PSW) using the composite interval mapping (CIM) method while no QTLs were detected in the case of four traits (i.e., FM, FD, FW, and SSC) (**Figure 5**; **Table 4**). Two QTLs were detected for EC on chromosomes 3 and 4, while one was detected for FL on chromosome 7, one was detected for FT on chromosome 10, two were detected for FC on chromosomes 4 and 8, one was detected for ND on chromosome 2, one was detected for NT on chromosome 2, one was detected for PSW on chromosome 2, and one was detected for the RLD on chromosome 7. The average spacing between bins was enhanced to 2.2 and 4.5 cM, respectively, by randomly selecting 20% (504) and 10% (255) of the 2,493 total bins; the QTL for EC on chromosome 3 was not detected in either of the genetic maps based on these bin subsamples, while the QTL for FC on chromosome 4 was not detected by the 255 bins map.

**Table 4:** Quantitative trait loci (QTL) mapping results using composite interval mapping (CIM) and 2,493 bins.

Trait	Chr	Logarithm of odds (LOD)	Peak (cM)	95% credible interval (cM)		Nearest bin^a^	Flanking bins^a^		Additive effect^b^	PVE (%)^c^
EC	3	4.57	70.71	66.43	71.63	S3_27813199	S3_27435285	S3_28191793	0.6302	14.85
	4	11.27	4.59	0.00	6.00	S4_1583322	S4_205086	S4_1655078	-1.0580	39.8
FL	7	6.06	52	49	54.82	S7_22247752	S7_21673362	S7_22751211	1.0372	19.82
FT	10	6.47	7.54	5	9.59	S10_1291780	S10_771413	S10_1545821	-0.2770	15.29
FC	4	5.11	9.03	8	12	S4_1736057	S4_1664870	S4_1966752	-0.7697	19.75
	8	10.20	76.73	73.63	77.71	S8_29946807	S8_29681675	S8_30003393	-1.0594	36.57
ND	2	9.40	40.99	39	41.04	S2_17480256	S2_16415727	S2_17972200	-0.6432	29.32
NT	2	6.49	33.66	32.26	34.60	S2_11852937	S2_7481584	S2_14567314	-0.4776	24.34
PSW	2	6.19	35.00	34	38	S2_14567314	S2_11852937	S2_16194372	-0.5565	21.93
RLD	7	6.73	52.28	51	54.40	S7_22398054	S7_21928298	S7_22713003	0.0605	17.18

*^a^Recombination bin represented by the first SNP in each bin.*

*^b^Additive effect was positive when the “TARI-08874” allele increased the trait score and was negative when the “Bai-Li-Gua” allele increased trait score.*

*^c^PVE, percentage of variance explained by the QTL.*

Comparing the map based on all 2,493 bins with results just using the smaller subsets shows that average differences in LOD peak positions were 1.864 and 3.408 cM, respectively, while the largest difference was 5.455 cM for LOD peaks of flesh thickness between the maps constructed using all 2,493 bins and just 255 bins. In general, QTL intervals were smaller and values of variance explained by QTL were slightly larger when higher marker densities were employed (Supplementary Data Sheet 1).

## Discussion

We applied high throughput GBS technology in this study to identify SNPs in a population of recombinant inbred melon lines. Because the draft DHL92 genome is available (Garcia-Mas et al., 2012; Argyris et al., 2015), physical positions and order of SNPs can be directly assigned by aligning short reads to this draft, rather than constructing linkage groups and ordering them based on recombination frequency. Indeed, given the high density of ordered SNPs, positions of recombination breakpoints can be identified precisely, greatly enhancing the resolution of the resultant genetic map (Huang et al., 2009). The significant proportion of missing data that results because of low coverage sequencing of multiplexed samples can be accurately imputed with algorithms that use haplotypes where the SNP marker order is known. However, one key factor determining the accuracy of imputation is quality of the original data.

### Constructing a Genetic Map Using High Quality Markers

In our preliminary analysis of the initial GBS dataset prior to imputation, the groupings and ordering of 4,164 SNPs shared between the reference-based and MST-constructed *de novo* maps were identical, with the exception of 54 SNP markers that were assigned to the wrong linkage groups and a 1.1 Mb inverted segment on chromosome 1. This misallocation issue was easily resolved by inspecting the recombination frequencies of markers in a bi-parental cross-derived population, as misallocation can potentially identify a false positive QTL (Supplementary Figure S5). However, because the BLAST alignment of 54 misallocated markers shared a high degree of agreement with the physical positions determined by BWA, this suggests that misallocation was not due to the alignment algorithm or to the randomly assignment of multiple best hits. A similar example of misallocated markers was also reported in a GBS study on cabbage (Lee et al., 2016); in this case, misallocated markers were probably derived from repeat-rich regions where recombination is usually suppressed. Thus, by evaluating recombination rates across the whole genome subsequent to genetic map construction, our results demonstrate that misallocated markers were indeed located in, or near to, regions where there was a low rate of recombination (**Figure 4**). The similar pattern of recombination rate in chromosome 10 was also reported in the study published by Argyris et al. (2015). According to their BLAST analysis with centromere-specific repeats, the centromere of chromosome 10 was located near the end of chromosome, we suspect the pattern of recombination rate was potentially affected by the location of centromere.

It is possible that the inverted segment identified on chromosome 1 is a result of rearrangement in parental lines compared to the draft DHL92 genome. Spurious recombination events were generated at the junctions of this inverted segment before it was corrected, further affecting imputation efficiency and accuracy.

### Accuracy of SNPs Following Filtering and Imputation

It is worth noting that when the SNPs were filtered using high-quality flanking anchors, the number and quality of final markers could have been affected by the proportion of missing information in the original data set, the significance level of Fisher’s exact test, and the number of anchors tested. Because our main motivation for the development of high-density GBS markers is to improve the resolution of QTL mapping via increased marker density, some loci with slightly higher proportions of missing data were incorporated. However, following imputation, average missing data across the genome-wide 23,413 loci decreased to 1.04% while imputation accuracy was more than 99%; this result strongly suggests that imputation accuracy was not affected by the quality of our data. Indeed, imputation accuracy of the loci we tested shows that the FSFHap algorithm worked effectively across our dataset, consistent with the accuracy of 99% reported by Swarts et al. (2014) in their study of maize-nested association mapping RILs (**Table 1**).

As discussed, prior to the construction of a genetic map for the RILs, we used the sliding window method to remove potential genotyping errors which could lead to spurious double recombination events and an inflation of genetic distance. Because of the high marker density in our dataset, genotypic value can be inferred from neighboring markers if two are linked genetically. The sliding window method performs poorly at the far ends of chromosomes and near to recombination breakpoints because of absent or incorrect information from adjacent markers (van Os et al., 2005), thus genotyping errors in these regions can remain in the final data set. Because just 0.17% of our data was identified as genotyping error by the sliding window method, the probability of the removal of correct data points should be extremely low. Indeed, in theory, the impact of incorrect removal is further reduced by our iterative process of linkage map construction.

Our final filtered dataset included a large segment that comprised 937 markers in the center of chromosome 6, significantly skewed towards TARI-08874. Such segregation distortion on chromosome 6 was not reported by Wang et al. (2016) in their study of the F2 population derived from the same cross between the “TARI-08874” and “Bai-Li-Gua” cultivars, although they did detect a segregation distortion at the distal end of chromosome 1 with two SSR markers. This distortion might be the result of genetic mechanisms of sexual reproduction, including pollen abortion (Taylor and Ingvarsson, 2003), or unconscious selection during the development of the RI population.

### A High-density Genetic Map Built from High-Quality Imputed Data

In this study, we constructed a reference-based genetic spanning 1,088.3 cM on the basis of 22,933 imputed SNP markers. Our map is about 5% smaller than those previously reported by Diaz et al. (2011) and Argyris et al. (2015) who employed 580 and 1,592 markers, respectively. Similarly, the 626.1 cM genetic map reported by Wang et al. (2016) using the F2 population from the same parents was half the size of ours, a dramatic difference that is probably due to marker density and genetic makeup of the population. While just 75 polymorphic SSR markers were used in the study of Wang et al. (2016), a GBS approach was simultaneously able to identify high-density genome-wide SNPs in the mapped population.

Because melon netting on the external surface is an important trait affecting consumer acceptance in Asia, we used ND as an example to determine the performance of our linkage map based on QTL mapping. Employing the high-density map that used 22,933 markers, we detected a QTL for ND at 40.99 cM on chromosome 2. In this case, the size of the QTL was 2.04 cM from the genetic position 39 cM towards 41.04 cM, which corresponds to a 1.6 Mb physical distance from 16,415,727 bp toward 18,031,638 bp. On the basis of the integrated physical map on the Melonomics database^2^ (Diaz et al., 2015), this QTL is co-localized with another, “*ntd2.3”* (i.e., between 14,378,938 and 23,050,030 bp on chromosome 2), identified in a previous study (Harel-Beja et al., 2010), while a 7 cM QTL “*qND2”* that controls ND was identified in an F2 population (Wang et al., 2016). Comparing the size of the QTL detected in this study with “*ntd2.3”* (27 cM) (Harel-Beja et al., 2010) and “*qND2*” (7 cM) (Wang et al., 2016), our high-density marker map demonstrates significant potential for enhancing the resolution of QTL mapping.

A number of simulation studies have already suggested that precise QTL detection can be achieved by increasing marker density (Li et al., 2010; Stange et al., 2013). Our results using different densities show that the QTL intervals for ND were 2.0, 5.0, and 7.4 cM when using 2,493 bins, 504 bins, and 255 bins to construct maps, respectively, and supports the contention that the size of a QTL decreases as marker density increases. Nevertheless, one study has suggested that QTL detection power is not affected by increasing marker density when this is between 1 and 5 cM, but that it is affected by population size and QTL effect (Stange et al., 2013). Because we measured ND in 84 inbred lines in this study, our detection power was greatly limited by population size and thus just one major QTL was detected. It is clear that increasing marker density does improve precision of QTL detection.

In this study, we report the first use of GBS, a high throughput and cost-effective technology, to develop 22,933 high-quality SNP markers and to construct a high-density genetic map for a RIL population in melon. As a result of considerable data cleaning and the process of imputation, our final marker map outperformed any previously reported genetic map in the mapping resolution of QTL. Thus, we anticipate that the methodology presented here will facilitate the future development of GBS markers in melon and other plant species, enabling the development of high-quality genome-wide SNPs at low cost, as well as the application of a variety of genomics-assisted approaches (e.g., marker assisted and genomic selection) to accelerate melon breeding.

## Author Contributions

C-WC, Y-HW, and C-WT conceived and designed the experiments. Y-HW and C-WT performed the experiments. C-WC and C-WT analyzed the data. C-WC and C-WT wrote the manuscript.

## Conflict of Interest Statement

The authors declare that the research was conducted in the absence of any commercial or financial relationships that could be construed as a potential conflict of interest.

## Footnotes

1 http://www.melonomics.net

2 https://melonomics.net/

## References

[B1] Argyris J. M. Ruiz-Herrera A. Madriz-Masis P. Sanseverino W. Morata J. Pujol M. (2015). Use of targeted SNP selection for an improved anchoring of the melon (*Cucumis melo* L.) scaffold genome assembly. *BMC Genomics* 16:4 10.1186/s12864-014-1196-3 PMC4316794 25612459

[B2] Arumuganathan K. Earle E. D. (1991). Nuclear DNA content of some important plant species. *Plant Mol. Biol. Rep.* 9 208–218. 10.1007/BF02672069

[B3] Baudracco-Arnas S. Pitrat M. (1996). A genetic map of melon (*Cucumis melo* L.) with RFLP, RAPD, isozyme, disease resistance and morphological markers. *Theor. Appl. Genet.* 93 57–64. 10.1007/BF00225727 24162199

[B4] Bielenberg D. G. Rauh B. Fan S. Gasic K. Abbott A. G. Reighard G. L. (2015). Genotyping by sequencing for SNP-based linkage map construction and QTL analysis of chilling requirement and bloom date in peach [*Prunus persica* (L.) Batsch]. *PLOS ONE* 10:e0139406 10.1371/journal.pone.0139406 PMC4592218 26430886

[B5] Boissot N. Thomas S. Sauvion N. Marchal C. Pavis C. Dogimont C. (2010). Mapping and validation of QTLs for resistance to aphids and whiteflies in melon. *Theor. Appl. Genet.* 121 9–20. 10.1007/s00122-010-1287-8 20180095

[B6] Broman K. W. Wu H. Sen S. Churchill G. A. (2003). R/qtl: QTL mapping in experimental crosses. *Bioinformatics* 19 889–890. 10.1093/bioinformatics/btg112 12724300

[B7] Camacho C. Coulouris G. Avagyan V. Ma N. Papadopoulos J. Bealer K. (2009). BLAST+: architecture and applications. *BMC Bioinform.* 10:421 10.1186/1471-2105-10-421 PMC2803857 20003500

[B8] Churchill G. A. Doerge R. W. (1994). Empirical threshold values for quantitative trait mapping. *Genetics* 138 963–971. 7851788 10.1093/genetics/138.3.963 PMC1206241

[B9] Cuevas H. E. Staub J. E. Simon P. W. Zalapa J. E. (2009). A consensus linkage map identifies genomic regions controlling fruit maturity and beta-carotene-associated flesh color in melon (*Cucumis melo* L.). *Theor. Appl. Genet.* 119 741–756. 10.1007/s00122-009-1085-3 19551368

[B10] Cuevas H. E. Staub J. E. Simon P. W. Zalapa J. E. McCreight J. D. (2008). Mapping of genetic loci that regulate quantity of beta-carotene in fruit of US Western Shipping melon (*Cucumis melo* L.). *Theor. Appl. Genet.* 117 1345–1359. 10.1007/s00122-008-0868-2 18773190

[B11] Deleu W. Esteras C. Roig C. González-To M. Fernández-Silva I. Gonzalez-Ibeas D. (2009). A set of EST-SNPs for map saturation and cultivar identification in melon. *BMC Plant Biol.* 9:90 10.1186/1471-2229-9-90 PMC2722630 19604363

[B12] Diaz A. Fergany M. Formisano G. Ziarsolo P. Blanca J. Fei Z. (2011). A consensus linkage map for molecular markers and quantitative trait loci associated with economically important traits in melon (*Cucumis melo* L.). *BMC Plant Biol.* 11:111 10.1186/1471-2229-11-111 PMC3163537 21797998

[B13] Diaz A. Forment J. Argyris J. M. Fukino N. Tzuri G. Harel-Beja R. (2015). Anchoring the consensus ICuGI genetic map to the melon (*Cucumis melo* L.) genome. *Mol. Breed.* 35:188 10.1007/s11032-015-0381-7

[B14] Díaz A. Zarouri B. Fergany M. Eduardo I. Álvarez J. M. Picó B. (2014). Mapping and introgression of QTL involved in fruit shape transgressive segregation into ‘Piel de Sapo’ melon (*Cucucumis melo* L.). *PLoS ONE* 9:e104188 10.1371/journal.pone.0104188 PMC4134209 25126852

[B15] Edae E. A. Bowden R. L. Polan J. (2015). Application of population sequencing (POPSEQ) for ordering and imputing genotyping-by-sequencing markers in hexaploid wheat. *G3 (Bethesda)* 5 2547–2553. 10.1534/g3.115.020362 26530417 PMC4683627

[B16] Elshire R. J. Glaubitz J. C. Sun Q. Poland J. A. Kawamoto K. Buckler E. S. (2011). A robust, simple genotyping-by-sequencing (GBS) approach for high diversity species. *PLoS ONE* 6:e19379 10.1371/journal.pone.0019379 PMC3087801 21573248

[B17] Fang L. Yong X. Yue Z. Di C. JianMing F. ShaoGui G. (2009). Construction of permanent genetic map and comparative analysis of Xinjiang Hami melon [*Cucumis melo* L. ssp. melo convar. ameri (Pang.) Greb.]. *Acta Hortic. Sin.* 36 1767–1774.

[B18] FAOSTAT (2013). *Food and Agriculture Organization of the United Nations.* Available at: http://faostat3.fao.org/

[B19] Fernandez-Silva I. Eduardo I. Blanca J. Esteras C. Picó B. Nuez F. (2008). Bin mapping of genomic and EST-derived SSRs in melon (*Cucumis melo* L.). *Theor. Appl. Genet.* 118 139–150. 10.1007/s00122-008-0883-3 18806992

[B20] Fernandez-Silva I. Moreno E. Essafi A. Fergany M. Garcia-Mas J. Martín-Hernandez A. M. (2010). Shaping melons: agronomic and genetic characterization of QTLs that modify melon fruit morphology. *Theor. Appl. Genet.* 121 931–940. 10.1007/s00122-010-1361-2 20506012

[B21] Fukino N. Ohara T. Monforte A. J. Sugiyama M. Sakata Y. Kunihisa M. (2008). Identification of QTLs for resistance to powdery mildew and SSR markers diagnostic for powdery mildew resistance genes in melon (*Cucumis melo* L.). *Theor. Appl. Genet.* 118 165–175. 10.1007/s00122-008-0885-1 18797839

[B22] Garcia-Mas J. Benjak A. Sanseverino W. Bourgeois M. Mir G. González V. M. (2012). The genome of melon (*Cucumis melo* L.). *Proc. Nat. Acad. Sci. U.S.A.* 109 11872–11877. 10.1073/pnas.1205415109 PMC3406823 22753475

[B23] Gardner K. M. Brown P. Cooke T. F. Cann S. Costa F. Bustamante C. (2014). Fast and cost-effective genetic mapping in apple using next-generation sequencing. *G3 (Bethesda)* 1681–1687. 25031181 10.1534/g3.114.011023 PMC4169160

[B24] Glaubitz J. C. Casstevens T. M. Lu F. Harriman J. Elshire R. J. Sun Q. (2014). TASSEL-GBS: a high capacity genotyping by sequencing analysis pipeline. *PLoS ONE* 9:e90346 10.1371/journal.pone.0090346 PMC3938676 24587335

[B25] Haley C. S. Knott S. A. (1992). A simple regression method for mapping quantitative trait loci in line crosses using flanking markers. *Heredity (Edinb.)* 69 315–324. 10.1038/hdy.1992.131 16718932

[B26] Harel-Beja R. Tzuri G. Portnoy V. Lotan-Pompan M. Lev S. Cohen S. (2010). A genetic map of melon highly enriched with fruit quality QTLs and EST markers, including sugar and carotenoid metabolism genes. *Theor. Appl. Gen*et. 121 511–533. 10.1007/s00122-010-1327-4 20401460

[B27] Huang X. Feng Q. Qian Q. Zhao Q. Wang L. Wang A. (2009). High-throughput genotyping by whole-genome resequencing. *Genome Res.* 19 1068–1076. 10.1101/gr.089516.108 19420380 PMC2694477

[B28] Huang Y. F. Poland J. A. Wight C. P. Jackson E. W. Tinker N. A. (2014). Using genotyping-by-sequencing (GBS) for genomic discovery in cultivated oat. *PLoS ONE* 9:e102448 10.1371/journal.pone.0102448 PMC4105502 25047601

[B29] Jeffrey C. (1980). A review of the Cucurbitaceae. *Bot. J. Linn. Soc.* 81 233–247. 10.1111/j.1095-8339.1980.tb01676.x

[B30] Kerje T. Grum M. (2000). The origin of melon, *Cucumis melo*: a review of the literature. *Acta Hortic.* 510 37–44.

[B31] Lee J. Izzah N. K. Choi B.-S. Joh H. J. Lee S.-C. Perumal S. (2016). Genotyping-by-sequencing map permits identification of clubroot resistance QTLs and revision of the reference genome assembly in cabbage (*Brassica oleracea* L.). *DNA Res.* 23 29–41. 10.1093/dnares/dsv034 26622061 PMC4755525

[B32] Lester G. (1997). Melon (*Cucumis melo* L.) fruit nutritional quality and health functionality. *Horttechnology* 7 222–227.

[B33] Li H. Hearne S. Banziger M. Li Z. Wang J. (2010). Statistical properties of QTL linkage mapping in biparental genetic populations. *Heredity (Edinb.)* 105 257–267. 10.1038/hdy.2010.56 20461101

[B34] McCallum S. Graham J. Jorgensen L. Rowland L. J. Bassil N. V. Hancock J. F. (2016). Construction of a SNP and SSR linkage map in autotetraploid blueberry using genotyping by sequencing. *Mol. Breed.* 36 1–24. 10.1007/s11032-016-0443-5

[B35] Oliver M. Garcia-Mas J. Cardús M. Pueyo N. López-Sesé A. I. Arroyo M. (2001). Construction of a reference linkage map for melon. *Genome* 44 836–845. 10.1139/g01-073 11681608

[B36] Périn C. Hagen L. S. Giovinazzo N. Besombes D. Dogimont C. Pitrat M. (2002a). Genetic control of fruit shape acts prior to anthesis in melon (*Cucumis melo* L.). *Mol. Genet. Genomics* 266 933–941. 10.1007/s00438-001-0612-y 11862487

[B37] Périn C. Hagen S. De Conto V. Katzir N. Danin-Poleg Y. Portnoy V. (2002b). A reference map of *Cucumis melo* based on two recombinant inbred line populations. *Theor. Appl. Genet.* 104 1017–1034. 10.1007/s00122-002-0864-x 12582608

[B38] Pitrat M. (2008). “Vegetables I: Asteraceae, Brassicaceae, Chenopodicaceae, and Cucurbitaceae,” in *Melon*, eds Prohens J. Nuez F. (New York, NY: Springer), 283–315.

[B39] Ramamurthy R. K. Waters B. M. (2015). Identification of fruit quality and morphology QTLs in melon (*Cucumis melo*) using a population derived from flexuosus and cantalupensis botanical groups. *Euphytica* 204 163–177. 10.1007/s10681-015-1361-z

[B40] Sebastian P. Schaefer H. Telford I. R. H. Renner S. S. (2010). Cucumber (*Cucumis sativus*) and melon (*C. melo*) have numerous wild relatives in Asia and Australia, and the sister species of melon is from Australia. *Proc. Natl. Acad. Sci. U.S.A.* 107 14269–14273. 10.1073/pnas.1005338107 20656934 PMC2922565

[B41] Spindel J. Wright M. Chen C. Cobb J. Gage J. Harrington S. (2013). Bridging the genotyping gap: using genotyping by sequencing (GBS) to add high-density SNP markers and new value to traditional bi-parental mapping and breeding populations. *Theor. Appl. Genet.* 126 2699–2716. 10.1007/s00122-013-2166-x 23918062

[B42] Stange M. Utz H. F. Schrag T. A. Melchinger A. E. Würschum T. (2013). High-density genotyping: an overkill for QTL mapping? Lessons learned from a case study in maize and simulations. *Theor. Appl. Genet.* 126 2563–2574. 10.1007/s00122-013-2155-0 23860723

[B43] Stepansky A. Kovalski I. Perl-Treves R. (1999). Intraspecific classification of melons (*Cucumis melo* L.) in view of their phenotypic and molecular variation. *Plant Syst. Evol.* 217 313–332. 10.1007/BF00984373

[B44] Swarts K. Li H. Romero Navarro J. A. An D. Romay M. C. Hearne S. (2014). Novel Methods to optimize genotypic imputation for low-coverage, next-generation sequence data in crop plants. *Plant Genome* 7. 10.3835/plantgenome2014.05.0023

[B45] Taylor D. R. Ingvarsson P. K. (2003). Common features of segregation distortion in plants and animals. *Genetica* 117 27–35. 10.1023/A:1022308414864 12656570

[B46] Taylor J. Butler D. (2015). *ASMap: Linkage Map Construction using the MSTmap Algorithm (Version R package version 0.4-5).* Available at: http://CRAN.R-project.org/package=ASMap

[B47] Tzuri G. Zhou X. Chayut N. Yuan H. Portnoy V. Meir A. (2015). A ‘golden’ SNP in CmOr governs the fruit flesh color of melon (*Cucumis melo*). *Plant J.* 82 267–279. 10.1111/tpj.12814 25754094

[B48] van Os H. Stam P. Visser R. G. F. van Eck H. J. (2005). SMOOTH: a statistical method for successful removal of genotyping errors from high-density genetic linkage data. *Theor. Appl. Genet.* 112 187–194. 10.1007/s00122-005-0124-y 16258753

[B49] Wang Y.-H. Wu D.-H. Huang J.-H. Tsao S.-J. Hwu K.-K. Lo H.-F. (2016). Mapping quantitative trait loci for fruit traits and powdery mildew resistance in melon (*Cucumis melo*). *Bot. Stud.* 57 1–12. 10.1186/s40529-016-0130-1 PMC5430578 28597428

[B50] Wu Y. Bhat P. R. Close T. J. Lonardi S. (2008). Efficient and accurate construction of genetic linkage maps from the minimum spanning tree of a graph. *PLoS Genet.* 4:e1000212 10.1371/journal.pgen.1000212 PMC2556103 18846212

[B51] Yu H. Xie W. Wang J. Xing Y. Xu C. Li X. (2011). Gains in QTL detection using an ultra-high density SNP map based on population sequencing relative to traditional RFLP/SSR markers. *PLoS ONE* 6:e17595 10.1371/journal.pone.0017595 PMC3048400 21390234

[B52] Zhang G. Ren Y. Sun H. Guo S. Zhang F. Zhang J. (2015). A high-density genetic map for anchoring genome sequences and identifying QTLs associated with dwarf vine in pumpkin (*Cucurbita maxima* Duch.). *BMC Genomics* 16:1101 10.1186/s12864-015-2312-8 PMC4690373 26704908

