# Supplementary material for: Genome-Wide Single Nucleotide Polymorphism Discovery and the Construction of a High-Density Genetic Map for Melon (Cucumis melo L.) Using Genotyping-by-Sequencing
Source: Front Plant Sci. 2017 Feb 6;8:125. doi: 10.3389/fpls.2017.00125 (PMC5292975; doi:10.3389/fpls.2017.00125)
Supplement: Supplementary file 1 [file Presentation_1.PPTX]

## Slide 1
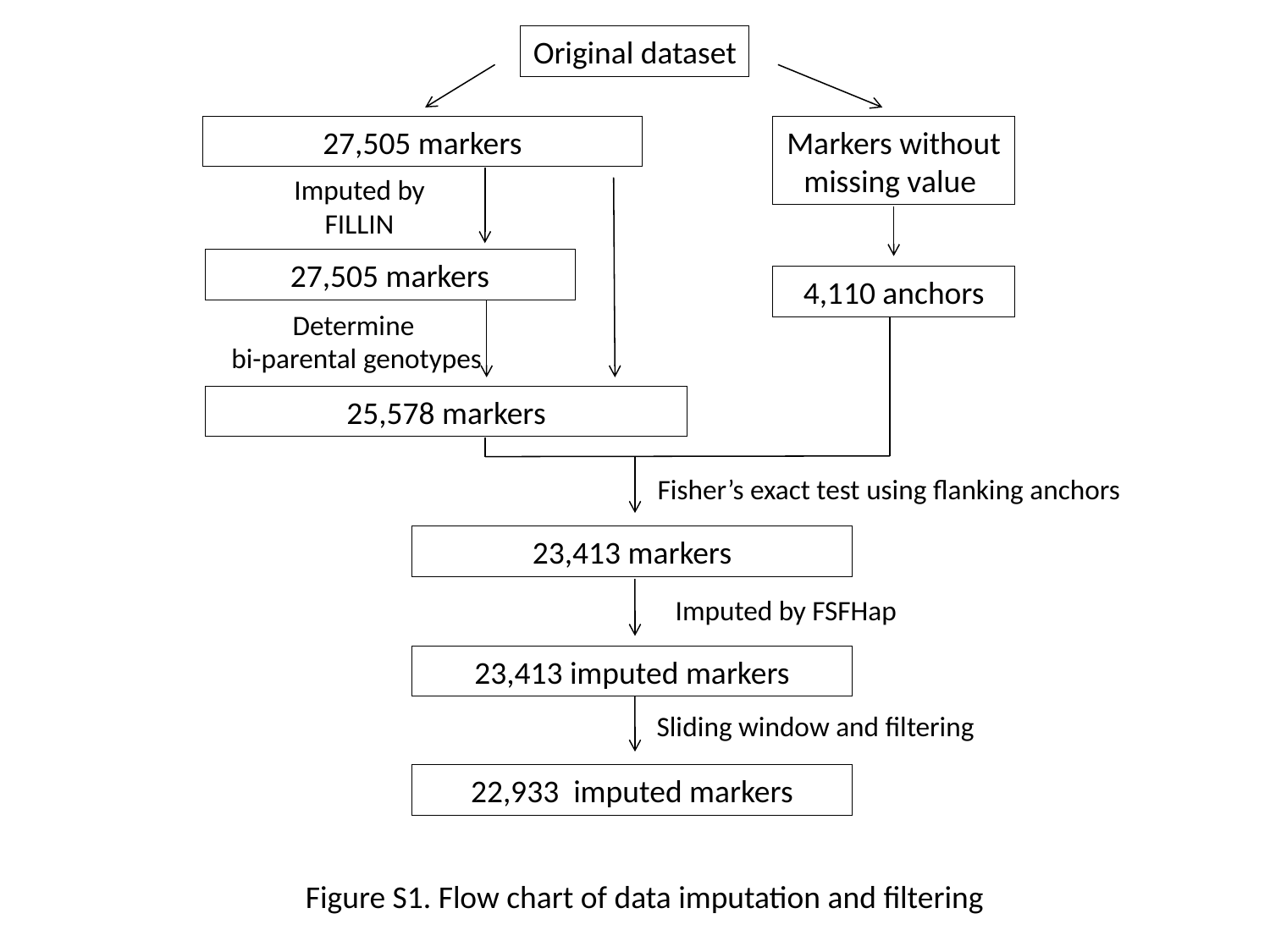

Original dataset
27,505 markers
Markers without missing value
Imputed by FILLIN
27,505 markers
4,110 anchors
Determine
bi-parental genotypes
25,578 markers
Fisher’s exact test using flanking anchors
23,413 markers
Imputed by FSFHap
23,413 imputed markers
Sliding window and filtering
22,933 imputed markers
Figure S1. Flow chart of data imputation and filtering
