# Supplementary material for: Genome-Wide Single Nucleotide Polymorphism Discovery and the Construction of a High-Density Genetic Map for Melon (Cucumis melo L.) Using Genotyping-by-Sequencing
Source: Front Plant Sci. 2017 Feb 6;8:125. doi: 10.3389/fpls.2017.00125 (PMC5292975; doi:10.3389/fpls.2017.00125)
Supplement: Supplementary file 2 [file Presentation_2.PPTX]

## Slide 1
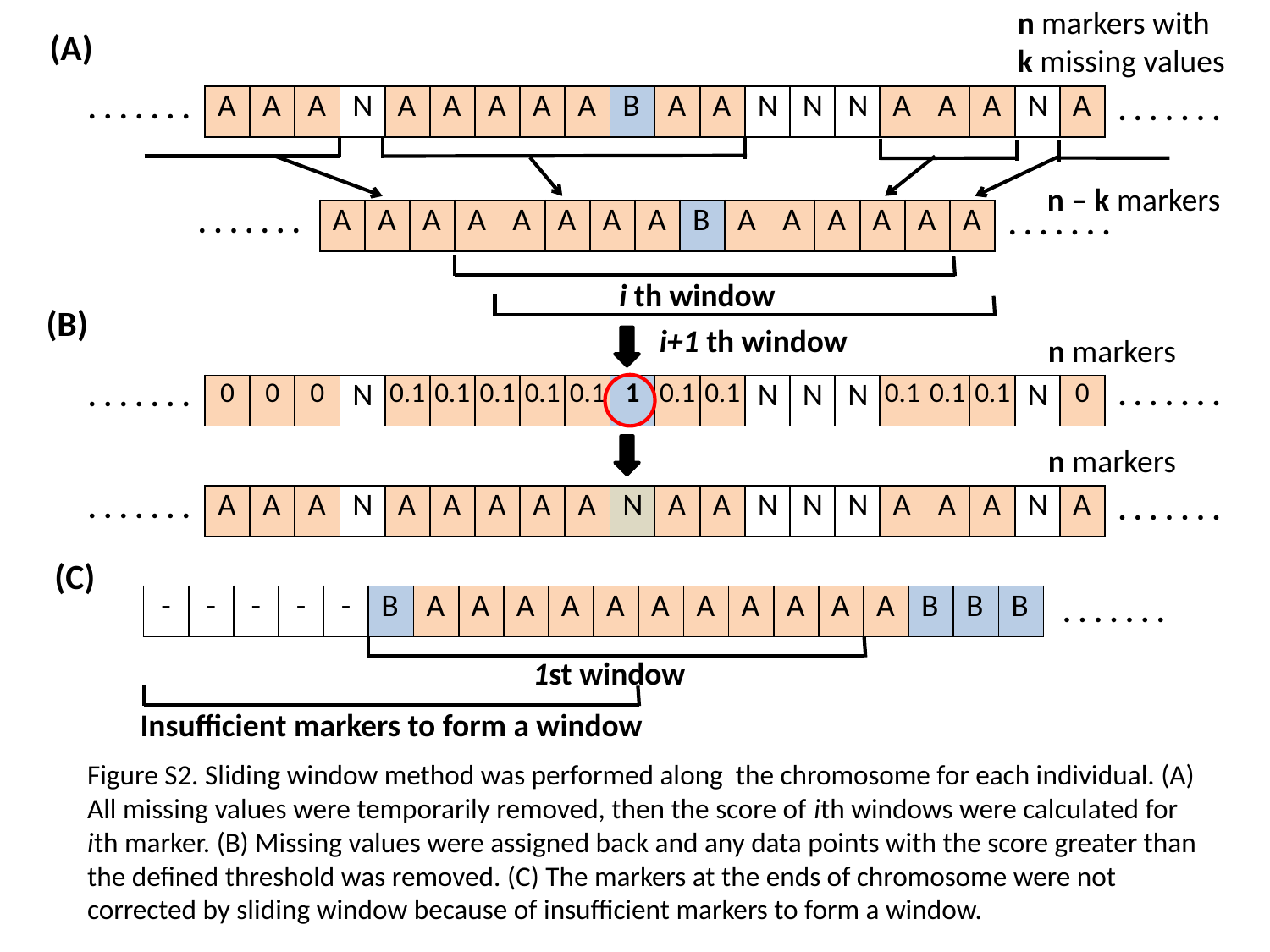

n markers with
k missing values
(A)
. . . . . . .
. . . . . . .
| A | A | A | N | A | A | A | A | A | B | A | A | N | N | N | A | A | A | N | A |
| --- | --- | --- | --- | --- | --- | --- | --- | --- | --- | --- | --- | --- | --- | --- | --- | --- | --- | --- | --- |
n – k markers
. . . . . . .
. . . . . . .
| A | A | A | A | A | A | A | A | B | A | A | A | A | A | A |
| --- | --- | --- | --- | --- | --- | --- | --- | --- | --- | --- | --- | --- | --- | --- |
i th window
(B)
i+1 th window
n markers
. . . . . . .
. . . . . . .
| 0 | 0 | 0 | N | 0.1 | 0.1 | 0.1 | 0.1 | 0.1 | 1 | 0.1 | 0.1 | N | N | N | 0.1 | 0.1 | 0.1 | N | 0 |
| --- | --- | --- | --- | --- | --- | --- | --- | --- | --- | --- | --- | --- | --- | --- | --- | --- | --- | --- | --- |
n markers
. . . . . . .
. . . . . . .
| A | A | A | N | A | A | A | A | A | N | A | A | N | N | N | A | A | A | N | A |
| --- | --- | --- | --- | --- | --- | --- | --- | --- | --- | --- | --- | --- | --- | --- | --- | --- | --- | --- | --- |
(C)
. . . . . . .
| B | A | A | A | A | A | A | A | A | A | A | A | B | B | B |
| --- | --- | --- | --- | --- | --- | --- | --- | --- | --- | --- | --- | --- | --- | --- |
| - | - | - | - | - |
| --- | --- | --- | --- | --- |
1st window
Insufficient markers to form a window
Figure S2. Sliding window method was performed along the chromosome for each individual. (A) All missing values were temporarily removed, then the score of ith windows were calculated for ith marker. (B) Missing values were assigned back and any data points with the score greater than the defined threshold was removed. (C) The markers at the ends of chromosome were not corrected by sliding window because of insufficient markers to form a window.
