# Supplementary material for: Genome-Wide Single Nucleotide Polymorphism Discovery and the Construction of a High-Density Genetic Map for Melon (Cucumis melo L.) Using Genotyping-by-Sequencing
Source: Front Plant Sci. 2017 Feb 6;8:125. doi: 10.3389/fpls.2017.00125 (PMC5292975; doi:10.3389/fpls.2017.00125)
Supplement: Supplementary file 3 [file Presentation_3.PPTX]

## Slide 1
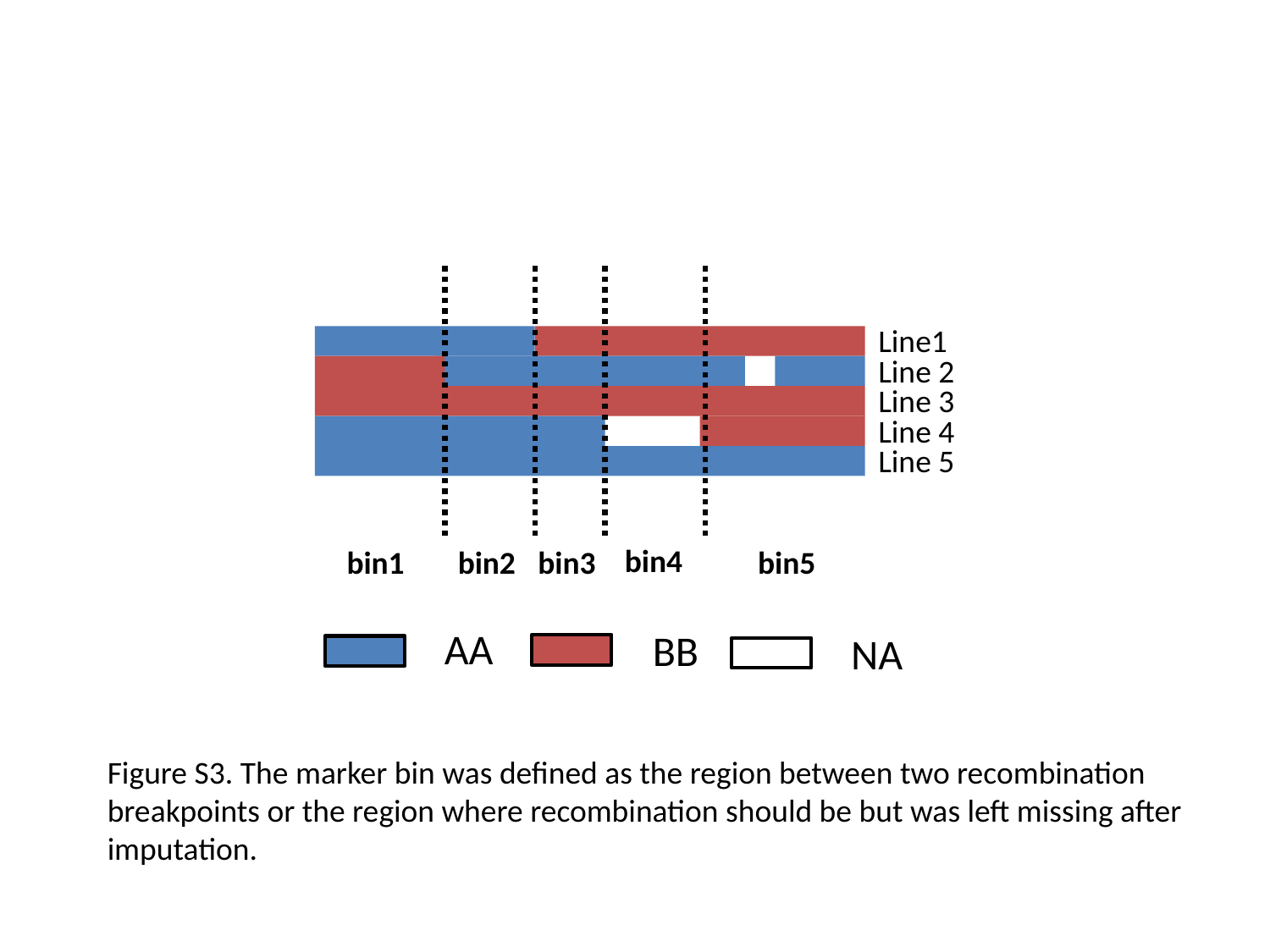

Line1
Line 2
Line 3
Line 4
Line 5
bin4
bin1
bin2
bin3
bin5
AA
BB
NA
Figure S3. The marker bin was defined as the region between two recombination breakpoints or the region where recombination should be but was left missing after imputation.
