# Supplementary figures and images for: Genome-Wide Single Nucleotide Polymorphism Discovery and the Construction of a High-Density Genetic Map for Melon (Cucumis melo L.) Using Genotyping-by-Sequencing
Source: Front Plant Sci. 2017 Feb 6;8:125. doi: 10.3389/fpls.2017.00125 (PMC5292975; doi:10.3389/fpls.2017.00125)

## Slide 1
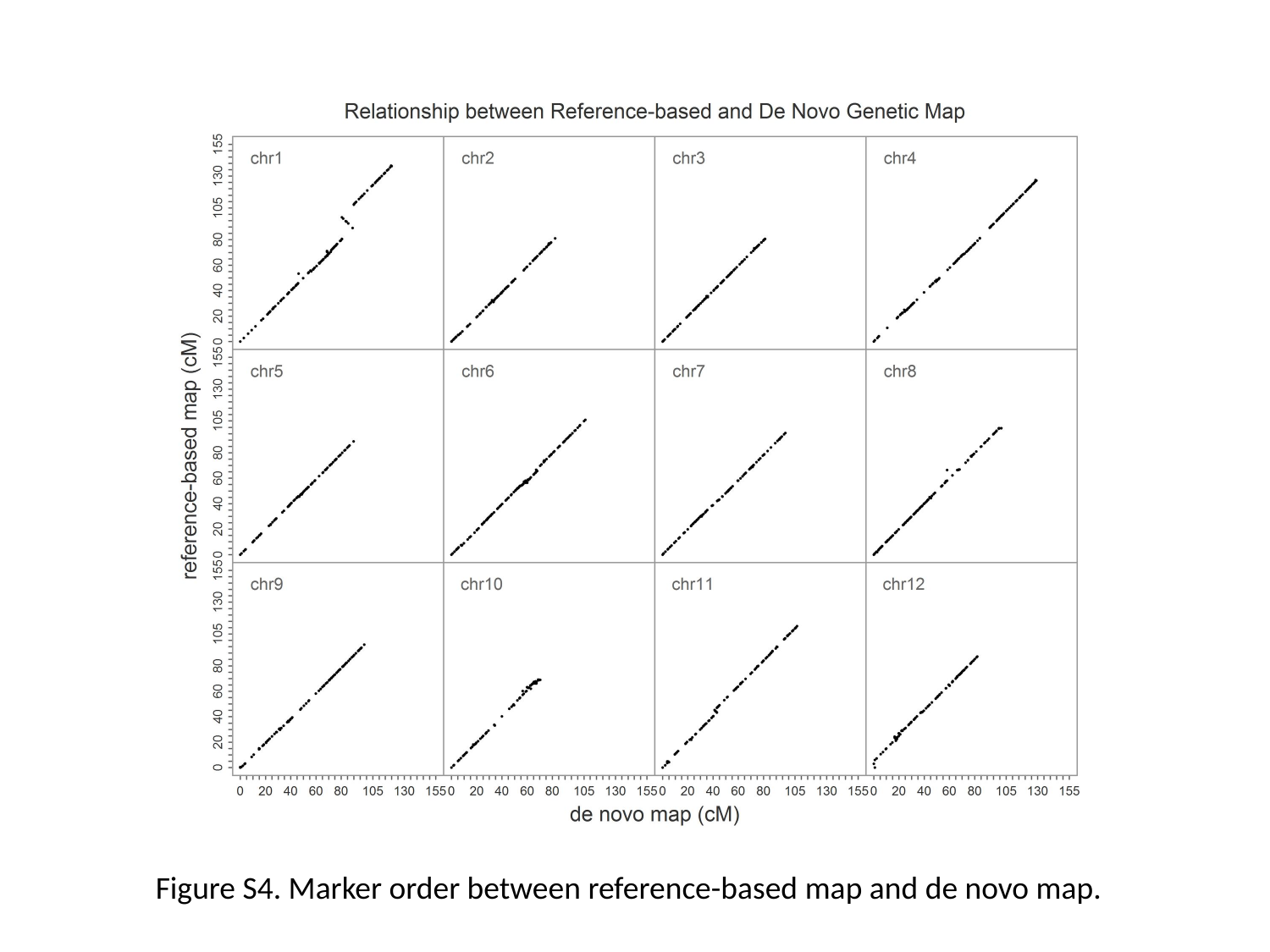

Figure S4. Marker order between reference-based map and de novo map.

Supplement: Supplementary file 4 [file Presentation_4.PPTX]
