# Supplementary material for: Genome-Wide Single Nucleotide Polymorphism Discovery and the Construction of a High-Density Genetic Map for Melon (Cucumis melo L.) Using Genotyping-by-Sequencing
Source: Front Plant Sci. 2017 Feb 6;8:125. doi: 10.3389/fpls.2017.00125 (PMC5292975; doi:10.3389/fpls.2017.00125)
Supplement: Supplementary file 5 [file Presentation_5.PPTX]

## Slide 1
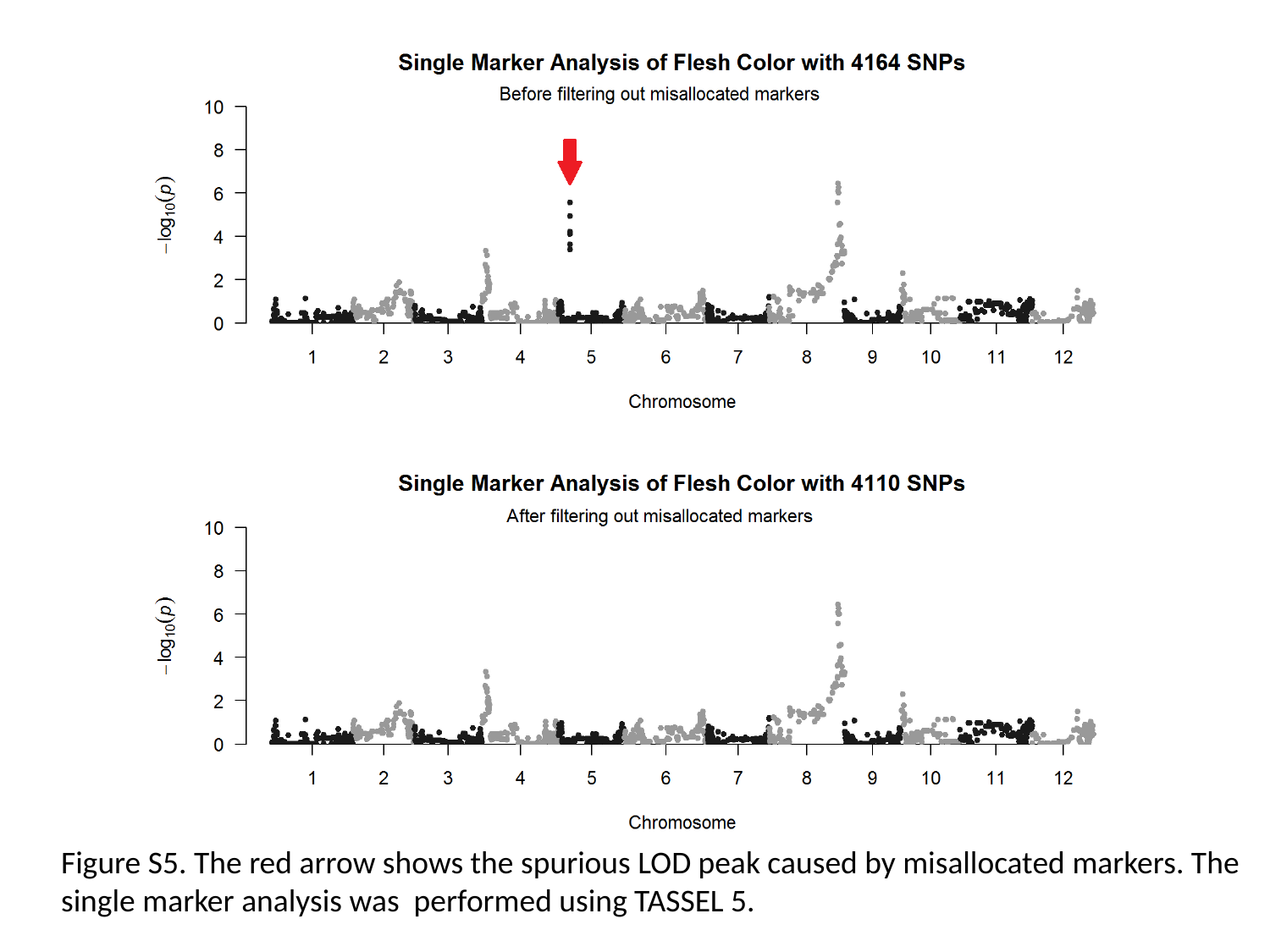

Figure S5. The red arrow shows the spurious LOD peak caused by misallocated markers. The single marker analysis was performed using TASSEL 5.
