# Supplementary material for: Genome-Wide Single Nucleotide Polymorphism Discovery and the Construction of a High-Density Genetic Map for Melon (Cucumis melo L.) Using Genotyping-by-Sequencing
Source: Front Plant Sci. 2017 Feb 6;8:125. doi: 10.3389/fpls.2017.00125 (PMC5292975; doi:10.3389/fpls.2017.00125)
Supplement: Supplementary file 6 [file Presentation_6.PPTX]

## Slide 1
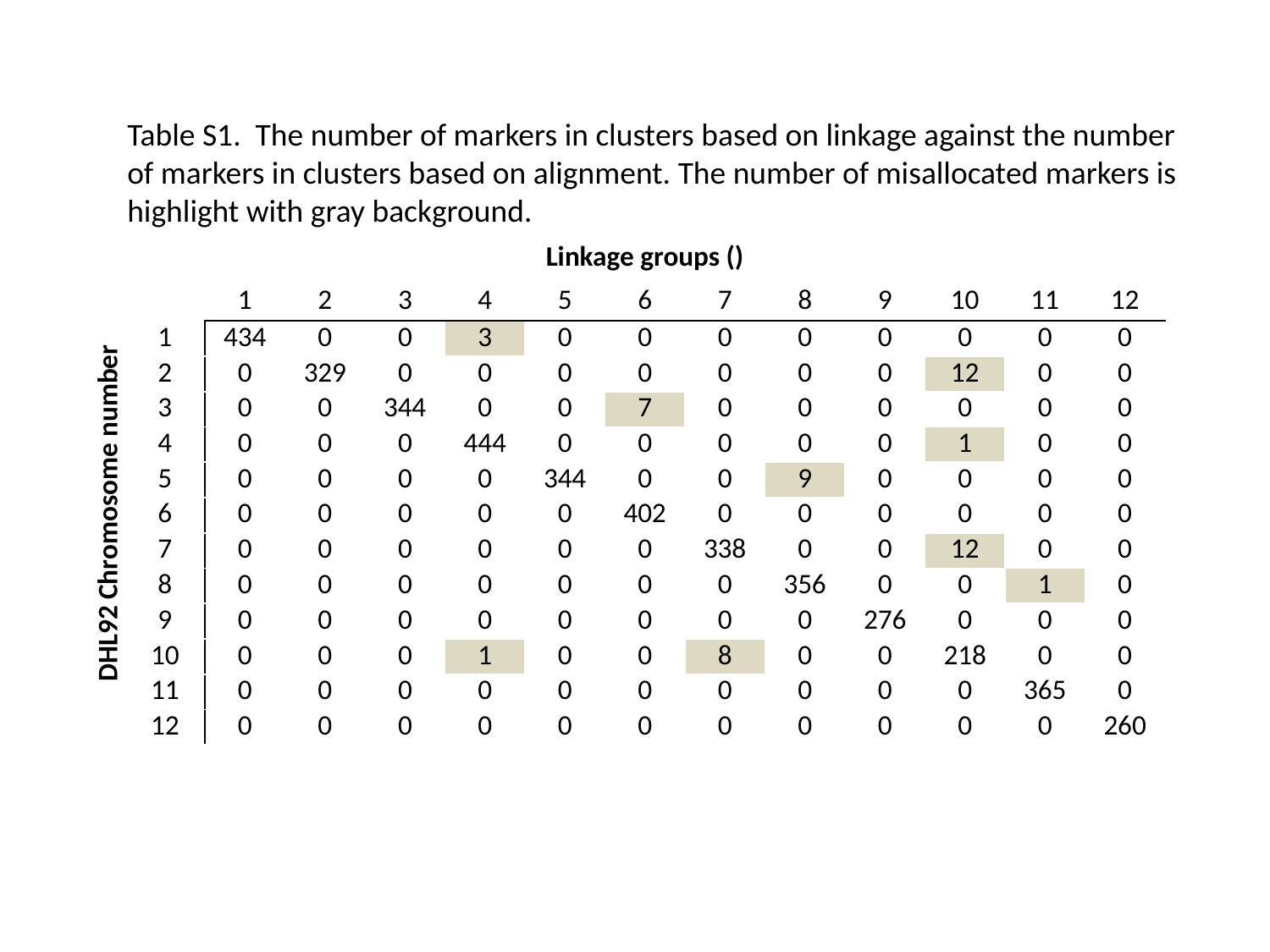

Table S1. The number of markers in clusters based on linkage against the number of markers in clusters based on alignment. The number of misallocated markers is highlight with gray background.
| | Linkage groups () | | | | | | | | | | | | |
| --- | --- | --- | --- | --- | --- | --- | --- | --- | --- | --- | --- | --- | --- |
| DHL92 Chromosome number | | 1 | 2 | 3 | 4 | 5 | 6 | 7 | 8 | 9 | 10 | 11 | 12 |
| | 1 | 434 | 0 | 0 | 3 | 0 | 0 | 0 | 0 | 0 | 0 | 0 | 0 |
| | 2 | 0 | 329 | 0 | 0 | 0 | 0 | 0 | 0 | 0 | 12 | 0 | 0 |
| | 3 | 0 | 0 | 344 | 0 | 0 | 7 | 0 | 0 | 0 | 0 | 0 | 0 |
| | 4 | 0 | 0 | 0 | 444 | 0 | 0 | 0 | 0 | 0 | 1 | 0 | 0 |
| | 5 | 0 | 0 | 0 | 0 | 344 | 0 | 0 | 9 | 0 | 0 | 0 | 0 |
| | 6 | 0 | 0 | 0 | 0 | 0 | 402 | 0 | 0 | 0 | 0 | 0 | 0 |
| | 7 | 0 | 0 | 0 | 0 | 0 | 0 | 338 | 0 | 0 | 12 | 0 | 0 |
| | 8 | 0 | 0 | 0 | 0 | 0 | 0 | 0 | 356 | 0 | 0 | 1 | 0 |
| | 9 | 0 | 0 | 0 | 0 | 0 | 0 | 0 | 0 | 276 | 0 | 0 | 0 |
| | 10 | 0 | 0 | 0 | 1 | 0 | 0 | 8 | 0 | 0 | 218 | 0 | 0 |
| | 11 | 0 | 0 | 0 | 0 | 0 | 0 | 0 | 0 | 0 | 0 | 365 | 0 |
| | 12 | 0 | 0 | 0 | 0 | 0 | 0 | 0 | 0 | 0 | 0 | 0 | 260 |
